# Different Functions of Recombinantly Expressed Domains of Tenascin-C in Glial Scar Formation

**DOI:** 10.3389/fimmu.2020.624612

**Published:** 2021-02-19

**Authors:** Dunja Bijelić, Marija Adžić, Mina Perić, Igor Jakovčevski, Eckart Förster, Melitta Schachner, Pavle R. Andjus

**Affiliations:** ^1^ Centre for Laser Microscopy, Faculty of Biology, Institute of Physiology and Biochemistry “Jean Giaja”, University of Belgrade, Belgrade, Serbia; ^2^ Institut für Neuroanatomie und Molekulare Hirnforschung, Ruhr-Universität Bochum, Bochum, Germany; ^3^ Keck Center for Collaborative Neuroscience and Department of Cell Biology and Neuroscience, Rutgers University, Piscataway, NJ, United States

**Keywords:** astrocyte, glial scar, microglia/macrophages, spinal cord injury, tenascin-C

## Abstract

Extracellular matrix glycoprotein tenascin-C (TnC) is highly expressed in vertebrates during embryonic development and thereafter transiently in tissue niches undergoing extensive remodeling during regeneration after injury. TnC’s different functions can be attributed to its multimodular structure represented by distinct domains and alternatively spliced isoforms. Upon central nervous system injury, TnC is upregulated and secreted into the extracellular matrix mainly by astrocytes. The goal of the present study was to elucidate the role of different TnC domains in events that take place after spinal cord injury (SCI). Astrocyte cultures prepared from TnC-deficient (TnC-/-) and wild-type (TnC+/+) mice were scratched and treated with different recombinantly generated TnC fragments. Gap closure, cell proliferation and expression of GFAP and cytokines were determined in these cultures. Gap closure *in vitro* was found to be delayed by TnC fragments, an effect mainly mediated by decreasing proliferation of astrocytes. The most potent effects were observed with fragments FnD, FnA and their combination. TnC-/- astrocyte cultures exhibited higher GFAP protein and mRNA expression levels, regardless of the type of fragment used for treatment. Application of TnC fragments induced also pro-inflammatory cytokine production by astrocytes *in vitro*. *In vivo*, however, the addition of FnD or Fn(D+A) led to a difference between the two genotypes, with higher levels of GFAP expression in TnC+/+ mice. FnD treatment of injured TnC-/- mice increased the density of activated microglia/macrophages in the injury region, while overall cell proliferation in the injury site was not affected. We suggest that altogether these results may explain how the reaction of astrocytes is delayed while their localization is restricted to the border of the injury site to allow microglia/macrophages to form a lesion core during the first stages of glial scar formation, as mediated by TnC and, in particular, the alternatively spliced FnD domain.

## Introduction

Spinal cord injury (SCI) is a severe neurological disorder with a limited hope for recovery, thus presenting a health care and socioeconomic problem. SCI is a two-step process, primary mechanical injury is followed by secondary inflammation and apoptosis through which existing injury spreads further into the surrounding tissue (1). The hallmark of these events is the formation of the glial scar with two distinct parts. The lesion core is formed by stromal fibroblasts and inflammatory immune cells, while hypertrophic astrocytes demarcate the lesion border (2). The role of the glial scar has been often discussed, once seen as a barrier to complete regeneration (3), but often regarded as important for axonal regrowth and as the source of growth factors and other permissive molecules (4). A majority of cellular functions, signal transduction, and tissue homeostasis are maintained by extracellular matrix components (ECM) making the ECM an interesting target for modulation of the outcome of injury. It is noteworthy in this context that the ECM glycoprotein tenascin-C (TnC) is strongly upregulated after injury of central nervous system (5).

Tenascin-C (TnC) is a large multimodular glycoprotein with a hexabrahion structure (6). Each arm contains four distinct domains: amino-terminal tenascin assembly (TA) domain, epidermal growth factor-like (EGFL) repeats, fibronectin type III (FnIII) domains, and a globular fibrinogen-homology domain (FG) located at the distal end. Among FnIII repeats, eight domains are constitutively expressed (Fn1-8), while nine are alternatively spliced (FnA1, FnA2, FnA3, FnA4, FnB, FnAD2, FnAD1, FnC, and FnD). Alternative RNA splicing yields various isoforms of TnC with different numbers of domains (7, 8). Via its structurally distinct domains and by variations in domains resulting from alternative splicing, TnC is predisposed to interact with different types of cellular receptors or components of the extracellular matrix, thereby generating an impressive functional diversity (9). In the central nervous system, TnC is widely expressed at early developmental stages (10), being mainly synthesized by immature astrocytes and radial glia during neuronal migration and differentiation (11). TnC stimulates astrocyte proliferation *in vitro* (12) and contributes to regenerative processes, such as peripheral nerve regeneration and wound healing in the brain (13–15). It also regulates the phenotype of cultured astrocytes *in vitro*, thus possibly contributing to astrocytic scar formation after spinal cord injury (16). TnC is also known to interact with other extracellular matrix components, including fibronectin (17, 18) and chondroitin sulfate proteoglycans (19, 20), relevant for regeneration after injury.

Recovery of locomotor functions after spinal cord injury in constitutively TnC-deficient (TnC-/-) mice was found to be reduced when compared with their wild-type (TnC+/+) littermates. Overexpression of the FnD domain of TnC in injured spinal cords improved regeneration (21). In TnC-/- mice synaptic rearrangements in the lumbar spinal cord and the H-reflex response were both attenuated after injury when compared with wild-type littermates. It is thus conceivable that TnC exerts its beneficial effects by modifying synaptic responses to injury (21). These findings are in agreement with previous observations that TnC enhances neurite outgrowth and supports neuronal survival (22, 23).

TnC is transiently expressed in acute inflammation and may contribute to chronic inflammation in pathological conditions (24). TnC also plays a role in temporal and spatial modulation of inflammation after SCI (25). Since TnC may promote axonal regrowth during acute inflammation, but also contribute to further damage during the chronic phase, investigation of individual TnC domains at specific times and in specific cell types is much needed. Since after SCI TnC is secreted mainly by astrocytes, we examined the impact of individual TnC fragments on astrocytic physiology *in vitro* and *in vivo*.

TNC-null mice were crucial for discovering the significance of heterophilic and homophilic TNC interactions in glial scar formation. The same mice were instrumental in the initial discovery of positive effects of TNC on regeneration after spinal cord injury (21). We now report on the dissection of functional TNC domains which may allow designing structures of therapeutic value. Results of our present study indicate that gap closure* in vitro *in an astrocyte scratch assay is delayed by TnC fibronectin-like fragments, mainly due to decreasing astrocyte proliferation. Fragments also upregulated mRNA levels of pro-inflammatory cytokines in astrocyte cell cultures. In spinal cord tissue, addition of TnC fragments immediately after SCI did not alter total cell proliferation rate. TnC, and in particular fragment FnD increased the numbers of activated microglia/macrophages 7 days after injury. Altogether results may explain how astrocyte functions are restricted to the border of the injury site to allow microglia/macrophages to form a lesion core during the first stages of glial scar formation through TnC, and in particular FnD.

## Materials and Methods

### Animals

Animals used in experiments were wild-type C57BL/6 (TnC+/+) mice and constitutively tenascin-C deficient (TnC-/-) mice inbred on the C57BL/6 background for more than 10 generations, and maintained in the Animal Facility of the Faculty of Biology, University of Belgrade. Animals were housed under standard conditions (21 ± 1°C, 50% humidity, 12:12 h light/dark cycle, water and food ad libitum). TnC-/- mice were derived from the original colony (26). All experimental procedures complied with the NIH Guide for Care and Use of Laboratory Animals (1985) and the European Communities Council Directive (86/609/EEC) and were approved by the Ethics Committee of the Faculty of Biology, University of Belgrade.

### Treatments

Five recombinantly expressed proteins representing different domains of TnC were studied. We used all alternatively spliced fragments and some of these that are present in all variants of the tenascin-C molecule. For injury experiments, we used the alternatively spliced FnD and FnA fragments, as they were shown to be the most promising ones in the *in vitro* experiments (23) and in a previous *in vivo* study (21). Alternatively spliced fragments are of special interest as they are upregulated upon injury (27).

Fibronectin type III-like repeats 6-8 (Fn 6-8) and epidermal growth factor-like repeats (EGFL) are constitutively expressed, whereas fibronectin type III-like repeats A, D, and C (FnA, FnD, FnC) are generated by alternative splicing. Fragments were generated as described (28). For *in vitro* experiments, treatment groups were labelled as follows: “SW” for the control group with only scratch wounding performed, “no SW” for non-injured cells, “EGFL”, “FnA”, “FnC”, “FnD”, “Fn6-8”, for groups in which the fragments were individually added after SW, and “Fn(D+A)”, “Fn(D+A+C)” for groups in which a combination of fragments was added. For *in vivo* experiments, all labels were the same except for the control group, which was named “Injury”, indicating that only compression spinal cord injury (SCI) was performed.

### Chemicals, Reagents, and Solutions

Acrylamide/Bis-acrylamide, ammonium persulfate (APS), bovine serum albumin (BSA), diethyl pyrocarbonate (DEPC), chloroform, EDTA, glucose, glycerol, Mowiol embedding medium, NP 40, paraformaldehyde (PFA), poly-L-lysine (PLL), sodium dodecyl sulphate (SDS), tetramethyl ethylenediamine (TEMED), Tris base, Triton™ X-100, trypsin, Tween 20, β-mercaptoethanol, NaCl, Na_2_HPO_4_, NaHCO_3_, NaOH, HCl, KH_2_PO_4_, KCl were from Sigma-Aldrich (St. Louis, Missouri, USA). Leibovitz’s L-15 medium, penicillin/streptomycin, foetal bovine serum (FBS), Dulbecco’s modified Eagle’s medium (DMEM) were from Gibco (Thermo Fisher Scientific, USA). TRIzol reagent, H_2_O, Power SYBR™ Green PCR Master Mix were from Invitrogen (Thermo Fisher Scientific, USA). High Capacity cDNA Reverse Transcription Kit was from Applied Biosystems (Thermo Fisher Scientific, USA). 4,6-Diamidino-2-phenylindole (DAPI) was from Molecular Probes (Thermo Fisher Scientific, USA). Protease/phosphatase inhibitor cocktail, Pierce micro BCA Protein Assay Kit, and PAGE ruler were from Thermo Fisher Scientific, USA. Clarity ECL Substrate was from BioRad Laboratories (Hercules, CA, USA). Ethanol, isopropanol, and methanol were from Zorka Pharma (Šabac, Serbia).

### Cortical Astrocyte Culture

Primary cell cultures were prepared as described (29). Briefly, for each genotype cortices from three mice, 0 to 2 days old, of both sexes were pooled. Tissue was mechanically dissociated and then centrifuged twice at 500xg for 5 min. Before the third centrifugation, the cell suspension was passed successively 3–5 times through 21G (ø 0.8 mm) and 23G (ø 0.6 mm) needles. All steps were performed in Leibowitz L-15 isolating medium supplemented with 100 IU/ml penicillin, 0.1 mg/ml streptomycin, and 0.1% BSA. Finally, cells were resuspended in growth medium (Dulbecco’s modified Eagle medium-low glucose, DMEM, supplemented with 10% foetal bovine serum, D-glucose to a final concentration of 25 mmol/L, 100 IU/ml penicillin, and 100 µg/ml streptomycin), seeded in a 60 mm Petri dish and maintained in a humidified atmosphere of 5% CO_2_/95% air at 37°C. The culture medium was replaced every 2 to 3 days. Upon reaching confluency, cells were trypsinized (0.25% trypsin and 0.02% EDTA) and seeded in new Petri dishes. Once confluence was reached again, cells were seeded according to the particular experimental design. Three cell cultures per genotype were prepared for all *in vitro* experiments.

### Scratch Wound Assay

For the scratch wound assay (SW), astrocytes were seeded in 35 mm Petri dishes at a density of 2×10^4^ cells/cm^2^ and maintained until complete confluency as described (30, 31). Monolayers were scratched with a sterile 200 µl pipette tip, followed by addition of fresh culture medium containing 10 µg/ml per TnC fragments. Three to four scratches were made per Petri dish. To ensure imaging of the same fields at different times, a straight line was drawn in the middle of the Petri dish bottom. For each SW, two areas, just above and below the line, were imaged using the AxioObserver A1 inverted microscope (Carl Zeiss GmbH, Germany), EM512 CCD (Digital Camera System, Evolve, Photometrics), and 10× (A-Plan) objective. Cells were imaged immediately after scratching and addition of fragments (0 h), and later at 6, 12, 24, and 48 h. For each image a gap border was selected and the gap area (µm^2^) was determined using the ImageJ software package. Relative wound closure was calculated with the formula:

$$\text{Relative}\;woundclosure=\left[A\left(t_{0}\right)-A\left(t\right)\right]/A\left(t_{0}\right)$$

where A represents wound area determined at a given time point t.

### Immunocytochemistry

For immunolabeling, cells were maintained on PLL-coated glass coverslips (ø 15 mm). Twenty-four h after scratching and treatment with TnC fragments, cells were fixed in 4% formaldehyde for 20 min at room temperature (RT). After several washes with phosphate-buffered saline (PBS), cells were permeabilized with 0.1% Triton in PBS for 15 min and blocked with a solution containing 5% BSA in PBS for 1 h, at RT. Following the overnight incubation with primary rabbit anti-Ki67 (1:500, Abcam, ab15580, RRID: AB_443209) or rabbit anti-GFAP (1:500, DAKO, Z0334, RRID: AB_10013382) antibodies in 1% BSA in PBS at 4°C, cells were washed and incubated with secondary donkey anti-rabbit AlexaFluor-555 antibodies for 2 h, at RT and in the dark (1:200, Invitrogen A-31572, RRID: AB_162543). Nuclei were stained with DAPI (1:4,000, for 15 min, at RT), and the glass coverslips were mounted on microscope slides with MOWIOL solution. Micrographs along the SW were acquired using AxioObserver A1 inverted microscope (Carl Zeiss GmbH, Germany), EM512 CCD (Digital Camera System, Evolve, Photometrics), and 10× (A-Plan) objective. Omission of primary antibodies did not show immunoreactivity. Images were quantified using ImageJ software. Proliferation rate was defined as the proportion of Ki67+ nuclei within the total number of DAPI+ nuclei. GFAP immunoreactivity was quantified and presented as the corrected total cell fluorescence (CTCF) of integrated density, calculated for each frame after using the “Rolling ball” background subtraction method in ImageJ software.

### Western Blot Analysis

Astrocytes seeded in 60 mm Petri dishes were scratched and treated with TnC fragments. Twenty-four h later, cultures were washed with pre-heated PBS, mechanically detached by scraping, collected in ice-cold PBS, and centrifuged for 5 min at 500×g. The pellet was resuspended in 500 µl of ice-cold RIPA lysis buffer, supplemented with 0.5% w/v protease inhibitor cocktail. Subsequently, lysates were centrifuged for 10 min at 10,000×g and 4°C. The supernatant was collected and the protein concentration was determined using the BCA protein assay kit, according to the manufacturer’s instruction. Samples (5 µg protein) were mixed with 6× Laemmle sample buffer (375 mM Tris-HCl, pH 6.8, 12% SDS, 60% w/v glycerol, and 0.03% bromophenol blue). Proteins were resolved on 12% SDS-PAGE gels and electrotransferred to a PVDF support membrane (Immobilon-P transfer membrane, Millipore, Merck, Germany). Membranes were blocked with 5% BSA in Tris buffered saline/Tween 20 (TBST) and incubated overnight at 4°C with primary rabbit anti-GFAP antibodies (1:7,000, DAKO, Z0334, RRID: AB_10013382), followed by a 2 h incubation with secondary HRP-conjugated donkey anti-rabbit antibody (1:10,000, Santa Cruz, sc-2305, AB_641180). Bands were visualized with ECL solution and Chemi Doc-It imaging system (UVP, Upland, CA, USA). Membranes were then subjected to Abcam mild stripping protocol: two times in stripping buffer for 10 min, two times in PBS for 10 min, and two times in TBST for 5 min. Membranes were blocked as above-mentioned, incubated with mouse anti-β-actin (1:1,000, Santa Cruz, sc-47778, RRID: AB_2714189) overnight at 4°C, followed by secondary HRP-conjugated donkey anti-mouse antibody (1:10,000, Santa Cruz, sc-2096, RRID: AB_641168). Bands were visualized as stated above, and quantified using ImageJ software. The measured optical density of GFAP immunoreactivity was normalized to the corresponding optical density of β-actin bands serving as a loading control.

### mRNA Isolation and Real-Time PCR

Astrocytes were seeded in 6-well plates at 2×10^4^ cells/cm^2^ density. After reaching confluence, the cultures were subjected to SW and treated with TnC fragments. Six hours later, sample lysates were collected using TRIzol reagent and the total RNA was subjected to phenol/chloroform extraction and ethanol precipitation (32). Since it has been shown that the median half-life of mRNA for all genes in mammals is up to 7 h, after which time mRNA is less stable/decays (33), we decided that 6 h would be the most appropriate time point to analyze the effects of fragments and also to not lose the information due to various signaling convergence and mRNA decay. The 6 h mRNA expression usually correlates with the protein abundance peak occurring 12–24 h later, which was confirmed in the present case. RNA concentrations were determined by measuring the absorbance at 260 nm and the purity was estimated from 260/280 nm and 260/230 nm ratios. For the synthesis of cDNA, 1 µg of total RNA was used. The real-time PCR reaction mixture contained 2 μl cDNA, 5 μl QTM SYBR Green PCR Master Mix, 0.5 μl of both reverse and forward primers (100 pmol/μl), and 2 μl RNase-free water. Amplification was carried out under the following conditions: 10 min of enzyme activation at 95°C, 40 cycles of 15 s denaturation at 95°C, 30 s annealing at 64°C, 30 s amplification at 72°C, and 5 s fluorescence measurements at 72°C. The amplification and product detection were carried out with QuantStudioTM 3 Real-Time PCR System (Applied Biosystems, Foster City, CA, USA). Relative gene expression is presented as a log_2_-fold change of mRNA expression. β-actin was used as a housekeeping molecule. Primer sequences are listed in **Table 1**.

**Table 1:** List of primer pairs for real-time PCR.

Target gene	Forward	Reverse
*GFAP*	CGGAGACGCATCACCTCTG	TGGAGGAGTCATTCGAGACAA
*TNF-α*	CTGAACTTCGGGGTGATCGG	GGCTTGTCACTCGAATTTTGAGA
*Il-1β*	AAAAGCCTCGTGCTGTCGGACC	TTGAGGCCCAAGGCCACAGGT
*β-actin*	GGGCTATGCTCTCCCTCAC	GATGTCACGCACGATTTCC

### Spinal Cord Injury

Compression spinal cord injury (SCI) was performed on 10- to 12-week-old mice as described (21). In short, before surgery, animals were anesthetized by intraperitoneal injections of ketamine and xylazine (100 and 5 mg/kg body weight, respectively, both from Sigma-Aldrich). Laminectomy was carried out at the T7–T9 level using mouse laminectomy forceps (Fine Science Tools, Foster City, California, USA). Then, the exposed spinal cord was compressed for 1 s using a custom-made device that consisted of watchmaker forceps mounted on a stereotaxic frame and driven by an electromagnetic device. Immediately after the injury, fragments FnD, FnA, their combination (300 µg/ml per treatment), or the vehicle control (0.9% NaCl in water) were injected into the lesion site. Injections into areas surrounding the injury site were also carried out 1 mm rostrally and caudally to the lesion site. Next, the skin was closed with a 3-0 silk suture (Ethicon, Somerville, New Jersey, USA). Mice were then kept on heating pads at 35°C for 24 h to prevent hypothermia and caged individually in a temperature-controlled room (22°C) on soft bedding with softened rodent chow and water within reach ad libitum. Bladders were manually voided twice a day. Animals were sacrificed 7 days later.

Four animals per group [vehicle control, FnA, FnD, Fn(D+A)] per each genotype (32 mice in total) underwent spinal cord injury. Criteria for the exclusion of mice from the study were, per ethical permit, weight loss of more than 20% over 24 h, passive position of animal in the cage, the lack of reaction to the experimenter, overall poor general condition as seen by the animal being immobile, cold with wet hair, and signs of severe urinary infection. Three animals were excluded for these reasons.

### Tissue Preparation and Immunohistochemistry

Mice were perfused transcardially under anesthesia with saline (0.9% NaCl) for 30 s, followed by perfusion with 4% formaldehyde in 0.1 M phosphate buffer for 10 min. The spinal cord was then removed through double laminectomy and placed in 4% formaldehyde for 2 h at 4°C. After post-fixation, tissue was cryoprotected in 0.1 M phosphate buffer supplemented with 30% sucrose at 4°C overnight and then frozen at -80°C until further use. Sagittal serial sections (25 μm thick) were cut using a LeicaCM 1850 cryostat (Leica Microsystems, Wetzlar, Germany), and sections were collected on SuperFrost^®^Plus slides (Menzel-Gläser, Braunschweig, Germany). After rehydration in PBS, sagittal spinal cord sections were covered with 0.1% glycine in PBS for 10 min at RT. Blocking with 10% bovine serum albumin (BSA) and 0.1% Triton X-100 was performed for 45 min at RT. Primary antibodies were diluted in 2% BSA in PBS and kept on the tissue sections overnight at 4°C. The following primary antibodies were used: mouse anti-GFAP (1:400, DAKO, Z0334), goat anti-Iba1 (1:300, Abcam) and rabbit anti-Ki67 (1:200, Abcam). After several washes in PBS, secondary antibodies diluted in PBS with 2% BSA were incubated for 2 h in the dark at RT. Secondary antibodies were donkey anti-mouse Alexa 488 (1:200, Invitrogen), donkey anti-goat Alexa 488 (1:200, Invitrogen), and donkey anti-rabbit Alexa 555 (1:200, Invitrogen). After washing with PBS, slides were incubated with TO-PRO 3 (1:40,000, Thermo Ficher Scientific T3605) to counterstain nuclei. Sections were rinsed with PBS and mounted in MOWIOL medium (Sigma Aldrich).

### Confocal Imaging, Analysis of Proliferation and GFAP Immunoreactivity in Tissue Sections

Cell proliferation and GFAP immunoreactivity at the injury site were analyzed in spinal cords of three animals per group [Injury only, Injury plus FnA, FnD, or Fn(D+A)] in both genotypes. Five sections per spinal cord were examined. A confocal laser microscope (LSM 510, Carl Zeiss, Jena, Germany) equipped with 488 nm Argon, 555 and 633 nm HeNe lasers was used for obtaining images. Z-stacks were obtained in the visible zone of the injury using oil-immersion Plan Neofluar 40x 1.3 NA objective. For proliferation rates, results were expressed as the portion of Ki67+ nuclei in total DAPI+ numbers in cell cultures or TO-PRO-3 stained nuclei in tissue sections using ImageJ (Rasband, W.S., ImageJ, National Institutes of Health, Bethesda, Maryland, USA, https://imagej.nih.gov/ij/, 1997-2018.). GFAP immunoreactivity was quantified and is presented as the corrected total cell fluorescence (CTCF) of integrated density, calculated for each frame after using the “Rolling ball” background subtraction method in ImageJ software.

### Stereological Analysis

The density of microglial cells in spinal cord tissue sections was obtained by stereological analysis as described (34). Counting was carried out with an Axio Imager. M1 microscope (Carl Zeiss) equipped with a motorized stage and a Stereo Investigator 9 software-controlled microscope system (MicroBrightField). First, a low-power magnification (10× objective) was used to outline the injury region and equally sized rostral and caudal regions next to it which extended for ≤1,500 μm on both sides of the injury site. In both genotypes, spinal cords from three animals were analyzed for each group (Injury, FnD). Six sections (25 μm, every 10 serial section) per spinal cord were investigated. Cells were counted based on Iba1 immunoreactivity and DAPI fluorescence and classified as activated microglia if they exhibited a polygonal shape or as resting if they showed a branched morphology. The following parameters were set: guard space depth, 2 μm, base and height of the dissector, 60x60 μm, and 10 μm; distance between the optical dissectors, 180 μm; objective 20× Plan-Neofluar 20×/0.50.

### Statistical Analysis

Two-way ANOVA was used to determine the effects of treatment and genotype regarding the examined parameters in cultures or tissue sections. Three-way ANOVA was used to analyze the effect of treatment, genotype and sampling position (injury, rostral or caudal) on the density of microglia. Statistically significant interactions, simple main effects, and pairwise comparisons were determined. All pairwise comparisons were run for each simple main effect with 95% confidence intervals and p-values Bonferroni-adjusted within each simple main effect. All computations were performed using the SPSS 20 software package (SPSS Inc., Chicago, IL, USA). Data from each experiment are summarized as a box and whisker plot and shown as mean ± SD throughout the Results section. Values for p within the range of 0.001–0.05 are given as precise numbers, whereas values lower than 0.001 are presented as <0.001.

## Results

### Tenascin-C Fragments Attenuate Gap Closure *In Vitro*

A scratch wound assay was performed in TnC+/+ and TnC-/- cortical astrocyte cultures to measure the response to mechanical injury (30). It is worth mentioning that mechanical stretching of an astrocyte monolayer is not the most adequate model for assaying glial scar formation since it lacks many components of *in vivo* injury (35). Unlike in tissues where astrocytes are confined to the lesion border, they are the only cell type in cultures.

**Figure 1:**
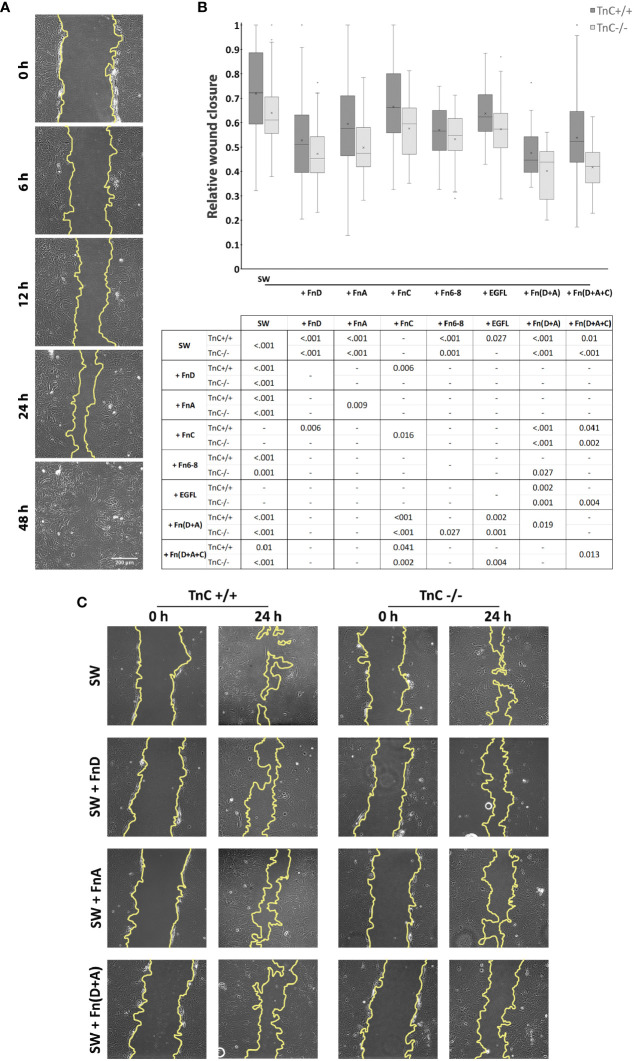
TnC fragments attenuate wound closure in cultured astrocytes. **(A)** Representative brightfield images of five time points of the gap area measured in the ImageJ program. **(B)** Relative wound closure is presented in a box-and-whisker plot indicating the impact of TnC fragments on gap closure in TnC+/+ and TnC-/- cortical astrocyte cultures after 24 h. Two-way ANOVA analysis shows no interaction between genotype and treatment, but both the effects of treatment and genotype were significantly different (*p* < 0.001). The most potent effect was produced by FnA, FnD, and combinations Fn(D+A) and Fn(D+A+C) in both genotypes. All pairwise significance comparisons are given in the table below the box-and-whisker plot. n = 3 independent astrocyte culture preparations. **(C)** Representative images of the gap at 0 and 24 h after scratching in the control group (SW) and groups treated with FnA, FnD, and Fn(D+A).

First, we assessed whether the rate of gap closure differs between the two genotypes upon the addition of different TnC fragments. Cells were monitored at 0, 6, 12, 24, and 48 h after scratching (see an example of ImageJ measurements in **Figure 1A**). Zero h served as a reference for closure calculation, and by 48 h all gaps were closed regardless of treatment. As described (36) and observed in our present study, the most informative time point is 24 h after scratching, allowing for evaluation of fragment application, astrocytic activation, and stable progression of the astrocytic front (**Figure 1B**). Quantifications of other time points with the exception of 0 and 48 h are given as stacked bar charts (**Supplementary Figure 1**). Representative images of the gap area of the same frame taken at 0 and 24 h in control group (SW) and groups treated with FnA, FnD, and Fn(D+A) are shown in **Figure 1C**.

Both the effects of treatment and genotype were significant (*p < 0.001*). The rate of injury closure reached the highest values in the control TnC+/+ group, with application of fragments retarding this process. Compared to the control group (SW), addition of FnA, FnD, Fn6-8, EGFL, Fn(D+A), and Fn(D+A+C) decreased the gap closure [p < 0.001 for FnA, FnD, Fn6-8, Fn(D+A); p = 0.027 for EGFL; p = 0.010 for Fn(D+A+C)]. Fragments FnC and EGFL did not affect gap closure. FnD decreased gap closure compared to FnC (*p* = 0.006). Combination Fn(D+A) slowed down the gap closure compared to FnC (*p* < 0.001) and EGFL (*p* < 0.001). Similarly, Fn(D+A+C) decreased gap closure compared to FnC (*p* = 0.041). In cultures of TnC-/- mice, where gap closure was most pronounced in the control group, fragments FnA, FnD, Fn6-8, Fn(D+A), and Fn(D+A+C) reduced gap closure [p < 0.001 for FnA, FnD, Fn(D+A), Fn(D+A+C); p = 0.010 for Fn6-8], while FnC and EGFL did not affect gap closure. The combined fragments Fn(D+A) also reduced gap closure when compared to FnC, Fn6-8, and EGFL (*p* < 0.001, *p* = 0.027, *p* < 0.001, respectively), while combination Fn(D+A+C) showed lower closure rates than FnC (*p* < 0.001) and EGFL (*p* = 0.004). In TnC+/+ cultures, gap closure was more pronounced than in TnC-/- cultures (*p* < 0.001). FnD and Fn6-8 attenuated gap closure in both genotypes, while FnA, FnC, Fn(D+A+C) attenuated gap closure in TnC-/- genotype (FnA: *p* = 0.009; FnC: *p* = 0.016; D+A+C: *p* = 0.013). Mean values ± standard deviations for each treatment and genotype are shown in **Table 2**.

**Table 2:** Mean value ± standard deviation for relative gap closure.

	SW	+EGFL	+FnA	+FnC	+FnD	+Fn6-8	+Fn(D+A)	+Fn(D+A+C)
TnC +/+	0.74 ± 0.19	0.64 ± 0.12	0.59 ± 0.19	0.66 ± 0.16	0.53 ± 0.19	0.57 ± 0.11	0.48 ± 0.11	0.54 ± 0.20
TnC -/-	0.64 ± 0.12	0.57 ± 0.13	0.50 ± .12	0.58 ± 0.11	0.47 ± 0.11	0.53 ± 0.11	0.40 ± 0.10	0.42 ± 0.09

### Tenascin-C Fragments Reduce Proliferation Rate *In Vitro*

**Figure 2:**
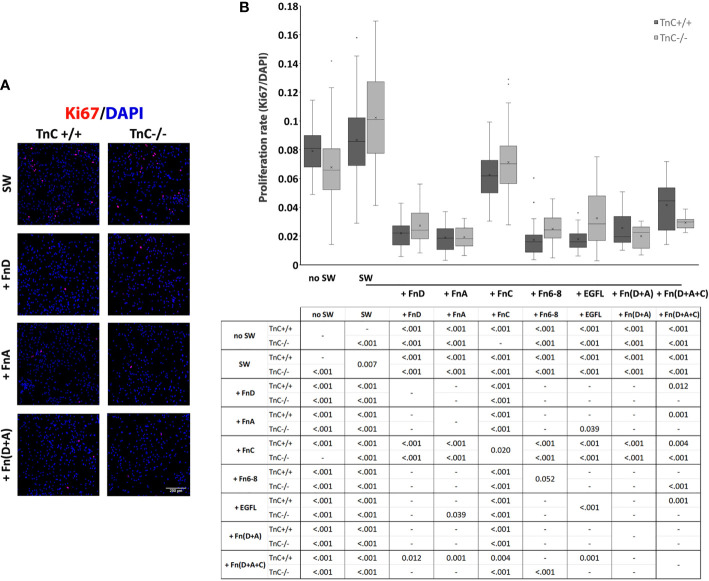
TnC fragments reduce proliferation in the astrocyte scratch wound assay. **(A)** Representative micrographs of Ki67+/DAPI+ immunofluorescence at 24 h after scratching in the control group and groups treated with FnA, FnD, and Fn(D+A); bar: 200 μm. **(B)** Proliferation was calculated as the number of Ki67+ nuclei compared to total DAPI+ nuclei. Results are presented as a box-and-whisker plot. Two-way ANOVA analysis shows a statistically significant interaction between the effects of genotype and treatment on cell proliferation rate (*p* = 0.005) with both the effects of genotype and treatment being significant (*p* = 0.040, *p* < 0.001, respectively). A statistically significant decrease in proliferation is seen in the presence of FnA, FnD, and Fn(D+A). All statistically significant pairwise comparisons are displayed below the box-and-whisker plot. n = 3 independent astrocyte cultures.

Migration and proliferation are two main processes that contribute to gap closure after scratching. Previous data show that the rate of astrocyte migration after scratching is the same as in non-injured, low density cultures used as a control group, whereas proliferation increases, making only proliferation an injury-specific response (30). On the basis of these observations, we assessed the proliferation rate (Ki67+/DAPI+ numbers) 24 h after scratching and application of fragments (**Figures 2A, B**). In this experiment another treatment group was added with no scratching and addition of fragments (no SW). There was a significant interaction between the effects of genotype and treatment on cell proliferation rate (*p* = 0.005) with both the effects of genotype and treatment being significant (*p* = 0.040, *p* < 0.001).

In TnC+/+ cultures the highest rate of proliferation was observed in the control SW group, while the addition of all fragments or their combinations decreased it [*p* < 0.001 for EGFL, FnA, FnC, FnD, Fn6-8, Fn(D+A), Fn(D+A+C)]. EGFL, FnA, FnD, Fn6-8, Fn(D+A), Fn(D+A+C) reduced proliferation compared to FnC [*p* < 0.001, except for Fn(D+A+C), *p* = 0.004]. Fn(D+A+C) led to a higher proliferation rate compared to FnD (*p* = 0.012), FnA (*p* = 0.001), Fn6-8 (*p* < 0.001), and EGFL (*p* = 0.001). The no SW group exhibited a higher proliferation rate than other treatments (*p* < 0.001), but was not different from the SW group.

Similarly, in the TnC-/- group the highest rate of proliferation was observed in the SW group, while the addition of all fragments or their combinations reduced it [*p* < 0.001 for EGFL, FnA, FnC, FnD, Fn6-8, Fn(D+A), Fn(D+A+C)]. Compared to FnC, the other treatments reduced the proliferation rate [EGFL, FnA, FnD, Fn6-8, Fn(D+A) and Fn(D+A+C), *p* < 0.001 for all]. Fragment FnA additionally reduced the proliferation rate compared to EGFL (*p* = 0.039). No SW group had a lower proliferation rate than the SW group (*p* < 0.001), but a higher proliferation rate than all other treatment groups (*p* < 0.001 for all) except for FnC. Comparisons within each treatment, between the two genotypes, showed a significant difference only in SW (*p* = 0.007), FnC (*p* = 0.020), Fn6-8 (*p* = 0.052), EGFL (*p* < 0.001) groups, where higher proliferation rates were observed in TnC-/- cultures. Mean values ± standard deviations for each treatment and genotype are shown in **Table 3**.

**Table 3:** Mean values ± standard deviations of proliferation rate in the astrocyte scratch wound assay.

	no SW	SW	+EGFL	+FnA	+FnC	+FnD	+Fn6-8	+Fn(D+A)	+Fn(D+A+C)
TnC +/+	0.079 ± 0.015	0.087 ± 0.026	0.018 ± 0.008	0.019 ± 0.010	0.062 ± 0.016	0.022 ± 0.010	0.017 ± 0.012	0.026 ± 0.013	0.042 ± 0.017
TnC-/-	0.078 ± 0.032	0.098 ± 0.032	0.032 ± 0.020	0.019 ± 0.007	0.071 ± 0.023	0.027 ± 0.011	0.025 ± 0.011	0.020 ± 0.008	0.029 ± 0.004

### GFAP Expression is Upregulated in Tenascin-C-/- Astrocyte Cultures Regardless of Fragment Addition

**Figure 3:**
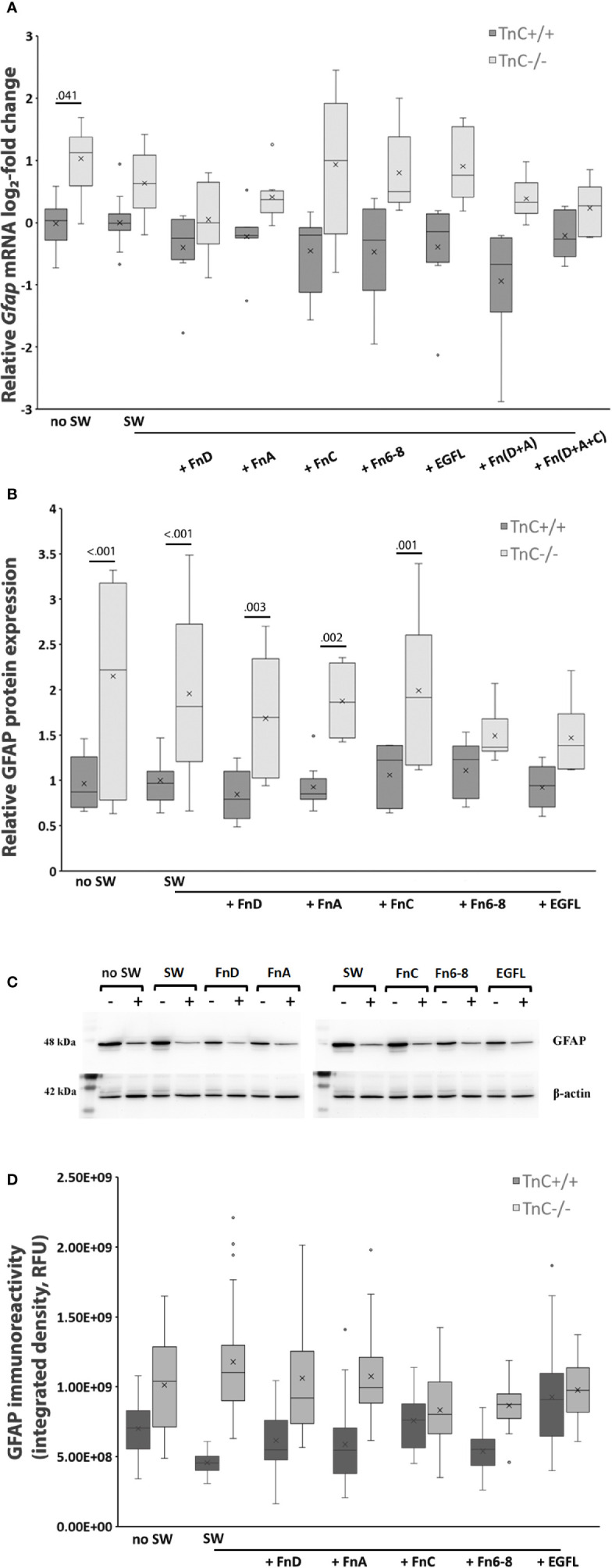
GFAP expression is upregulated in TnC-/- astrocyte cultures both in the absence and presence of fragments. **(A)** Relative log_2_-fold change of *Gfap* mRNA expression 6 h after scratching and fragment addition is shown in a box-and-whisker plot. Two-way ANOVA shows a genotype effect (*p* < 0.001). *Gfap* mRNA was significantly upregulated in TnC-/- no SW group compared to its TnC+/+ counterpart (*p* = 0.041). n = 4 independent astrocyte culture preparations. **(B)** Relative GFAP protein expression as estimated by Western blot analysis 24 h after scratching and application of fragments. Results are presented as box-and-whisker plot. Two-way ANOVA shows statistical significance of genotype (*p* < 0.001). GFAP is more expressed in the no SW group of TnC-/- versus TnC+/+ cultures (*p* < 0.001). Scratching alone and FnA, FnC, and FnD application lead to upregulation of GFAP protein levels in TnC-/- versus TnC+/+ astrocytes (*p* < 0.001; *p* = 0.003; *p* = 0.002: *p* = 0.001, respectively). n = 3 independent astrocyte culture preparations. **(C)** Representative images of Western blots for GFAP (48 kDa) and β‐actin (42 kDa). **(D)** GFAP immunoreactivity is presented as Integrated density box-and-whisker plot. n = 1 astrocyte culture.

GFAP, as a cytoskeletal marker protein of astrocytes, is upregulated after scratching (37). TnC also affects the levels of GFAP in lesion-activated astrocytes *in vitro* (38). Therefore, we examined *Gfap* mRNA (**Figure 3A**) and protein levels (**Figures 3B–D**) upon SW and treatment with TnC fragments (6 and 24 h, respectively).

Only the genotype`s effect on *Gfap* mRNA levels was significant (*p* < 0.001). *Gfap* mRNA expression levels in TnC -/- showed a log_2_-fold increase versus the TnC+/+ in the SW group. Compared to TnC+/+ cultures, upregulation of TnC-/- *Gfap* mRNA levels was most pronounced in the no SW group (TnC+/+ vs TnC-/-: 1.061 log_2_-fold change, *p* = 0.041) and in the FnC treatment group (TnC+/+ vs TnC-/-: 1.180 log_2_-fold change), while Fn(D+A) only tended to show upregulation (TnC+/+ vs TnC-/-: 0.680 log_2_-fold change, not significant).

GFAP protein expression was measured by Western blot analysis 24 h after SW and application of fragments (**Figure 3B**). Representative images of GFAP (48 kDa) and β‐actin (42 kDa) are in **Figure 3C**. Only the genotype`s effect on protein levels was significant (*p* < 0.001). GFAP was more expressed in the no SW group in TnC-/- cultures compared to TnC+/+ cultures (no SW: 2.15 ± 1.14; no SW: 0.97 ± 0.30, *p* < 0.001). Scratch wounding and treatments resulted in GFAP protein upregulation in TnC-/- cultures compared to the TnC+/+ cultures in the SW, FnD, FnA, and FnC groups (*p* < 0.001; *p* = 0.003; *p* = 0.002; *p* = 0.001, respectively). Immunostaining was performed to visually confirm Western blot GFAP protein expression levels (**Figure 3D**).

### Pro-Inflammatory Tnf-*α* and IL-1*β* Levels are Upregulated by FnD, FnA, Fn6-8, and EGFL in Both Genotypes

**Figure 4:**
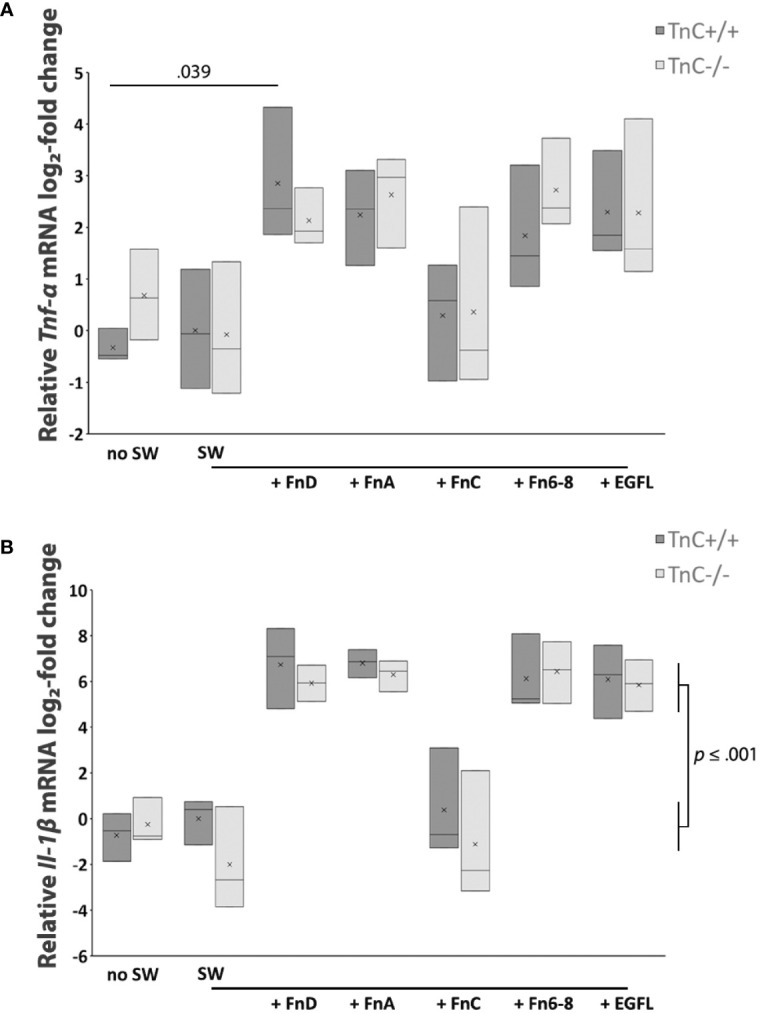
Pro-inflammatory cytokines TNF-α and IL-1β are upregulated by FnD, FnA, Fn6-8, and EGFL fragments in astrocyte cultures of both genotypes. **(A)** Relative log_2_-fold change of *Tnf-α* mRNA expression 6 h after scratching and fragment addition is presented in a box-and-whisker plot. Two-way ANOVA shows that only the treatment effect was significantly different (*p* < 0.001). In both TnC+/+ and TnC-/- genotypes, FnD, FnA, Fn6-8, and EGFL upregulate *Tnf-α* mRNA levels (~ 2 log_2_-fold change for all conditions) versus the no SW, SW, and FnC groups. Only the FnD fragment statistically upregulates *Tnf-α* mRNA levels versus the no SW in TnC+/+ cultures (*p* = 0.039). n = 3 independent astrocyte culture preparation. **(B)** Relative log_2_-fold change of *Il-1β* mRNA levels 6 h after scratching and fragment addition as presented in the box-and-whisker plot. Two-way ANOVA shows that only the treatment was statistically significant (*p* < 0.001). In TnC+/+ and TnC-/- cultures, addition of EGFL, FnA, FnD, and Fn6-8 upregulates *Il-1β* mRNA levels (~ 7 log_2_-fold change, *p* ≤ 0.001 for all conditions) versus levels of no SW, SW, and FnC. n = 3 independent astrocyte cultures.

It has been reported that TnC upregulates pro-inflammatory cytokine production in different cell types although there are no data available for astrocytes (39). Cytokine production in astrocyte cultures after scratching and addition of TnC fragments was tested in terms of mRNA levels of *Tnf-α*, *Il-1β*, *Il-6*, and *Il-10*, with *Il-6* and *Il-10* not being detectable. rtPCR results for *Tnf-α* mRNA levels showed that the treatment effect was significant (*p* < 0.001) (**Figure 4A**). In TnC+/+ cultures, addition of EGFL, FnA, FnD, and Fn6-8 fragments upregulated *Tnf-α* mRNA expression compared to SW, compared to the no SW group and compared to the FnC group. Similarly, in TnC-/- cultures, EGFL, FnA, FnD and Fn6-8 fragments increased *Tnf-α* mRNA levels compared to SW, no SW and FnC treatment group. In the case of *IL-1β* mRNA expression (**Figure 4B**) the effect of the treatment was significant (*p* < 0.001). Application of fragments EGFL, FnA, FnD and Fn6-8 upregulated *Il-1β* mRNA levels in TnC+/+ cultures compared to the SW group (*p* < 0.001 for FnA and FnD; *p* = 0.001 for EGFL and Fn6-8), no SW (*p* < 0.001 for EGFL, FnA, FnD, Fn6-8) and FnC (*p* = 0.001 for FnA and FnD; *p* = 0.003 for EGFL and Fn6-8). Similarly, in TnC-/- cultures, EGFL, FnA, FnD and Fn6-8 increased *Il-1β* mRNA expression when compared to SW (*p* = 0.001 for EGFL and FnA; *p* < 0.001 for FnD and Fn6-8), no SW (*p* < 0.001 for EGFL, FnA, FnD, Fn6-8) and FnC; (*p* < 0.001 for EGFL, FnA, FnD, Fn6-8). Mean values ± standard deviations for levels of proinflammatory cytokines TNF-α and IL-1β in astrocyte culture are presented in **Tables 4** and **5**, respectively.

**Table 4:** Mean values ± standard deviations of proinflammatory cytokine TNF-α in astrocyte cultures.

	no SW	SW	+EGFL	+FnA	+FnC	+FnD	+Fn6-8
TnC +/+	-0.33 ± 0.32	0.00 ± 1.16	2.29 ± 1.04	2.24 ± 0.92	0.29 ± 1.15	2.85 ± 1.30	1.84 ± 1.22
TnC-/-	0.68 ± 0.88	-0.08 ± 1.30	2.28 ± 1.60	2.63 ± 0.91	0.36 ± 1.79	2.13 ± 0.56	2.72 ± 0.88

**Table 5:** Mean values ± standard deviations of proinflammatory cytokine IL-1β levels in astrocyte cultures.

	no SW	SW	+EGFL	+FnA	+FnC	+FnD	+Fn6-8
TnC +/+	-0.73 ± 1.05	0.00 ± 0.99	6.09 ± 1.61	6.81 ± 0.61	0.37± 2.37	6.73 ± 1.78	6.12 ± 1.70
TnC-/-	-0.25 ± 1.02	-2.01 ± 2.27	5.84 ± 1.13	6.30 ± 0.68	-1.12 ± 2.81	5.92 ± 0.79	6.43 ± 1.35

### Tenascin-C Affects Astrocytes and Microglia/Macrophages in Injured Spinal Cords

*In vivo* glial scar formation was induced by compression SCI. Evaluation was performed 7 days after the injury when inflammation is in its early phase and thus can be modulated *via* different mechanisms (40).

**Figure 5:**
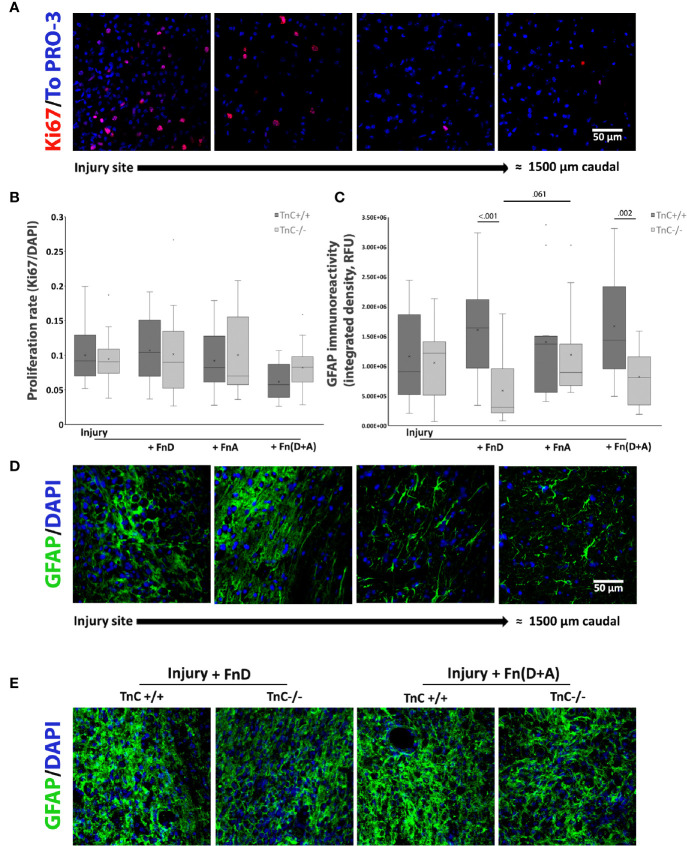
Proliferation and GFAP expression after spinal cord injury and TnC fragment application in TnC+/+ and TnC -/- mice. **(A)** Representative images of Ki67 and TO-PRO-3 immunostainings. More proliferating cells are present at the injury region, whereas a decrease in Ki67+ cells is observed further away from the injury site. Calibration bar for all panels: 50 μm. **(B)** Proliferation is calculated as the number of Ki67+ nuclei compared to TOPRO-3+ nuclei 7 days after injury. Results are presented as box-and-whisker plots. Even though the two-way ANOVA shows that the treatments’ overall effect was significant (*p* = 0.043), pairwise comparisons do not show significant differences. n = 3 animals per group for each genotype. **(C)** GFAP immunoreactivity at the injury region is presented as Integrated density in the box-and-whisker plot. Two-way ANOVA reveals a significant effect of genotype (*p* < 0.001) and significant interaction of genotype and treatment (*p* = 0.034). GFAP immunoreactivity is lower at 7 days after injury and application of FnD and Fn(D+A) in TnC-/- versus TnC+/+ mice. n = 3 animals per group for each genotype. **(D)** Representative images of GFAP and DAPI immunostaining reveal a change in the morphology of GFAP+ astrocytes at the injury region versus more caudal sites. Calibration bar for all panels: 50 μm. **(E)** GFAP and DAPI immunofluorescence at and around the injury site in the spinal cord of TnC+/+ and TnC-/- mice treated with FnD and Fn(D+A).

Proliferation *in vivo* was estimated in the same way as *in vitro* and in reference to all cell types in the injury region (**Figures 5A, B**). Ki67 immunolabeling indicated higher levels of proliferating cells within the region of injury and a decrease of such cells further away, in all groups (**Figure 5A**). Even though two-way ANOVA showed that the overall effect of fragment application was significant (*p* = 0.043), pairwise comparisons revealed no significant difference (**Figure 5B**).

Quantification of GFAP immunoreactivity (**Figure 5C**) in the injury region revealed a statistically significant effect of genotype (*p* < 0.001), as well as a statistically significant interaction of genotype and treatment effects (*p* = 0.034). Addition of fragment FnD to TnC-/- mice decreased GFAP immunoreactivity compared to FnD in TnC+/+ animals (*p* < 0.001). This effect was also observed with combined Fn(D+A) treatment (*p* = 0.002). Representative images of GFAP immunofluorescence at the injury site of spinal cord treated with FnD and Fn(D+A) fragments in both genotypes are presented in **Figure 5E**. In TnC-/- mice, although FnD reduced GFAP immunoreactivity compared to FnA, only a trend was observed (*p* = 0.061). Change in the morphology of GFAP positive astrocytes in the injury region compared to the more rostral or caudal regions in the spinal cord was also observed (**Figure 5D**). Astrocytes within the injury region formed a honeycomb-like structure, characteristic of the glial scar (41), whereas more distally they exhibited the fibrous quiescent phenotype (**Figure 5D**). Mean values ± standard deviations of GFAP immunoreactivity at the injury region are presented in **Table 6**.

**Table 6:** Mean values ± standard deviations of GFAP immunoreactivity at the injury site of the spinal cord.

	Injury	+FnA	+FnD	+Fn(D+A)
TnC +/+	1.16E^+06^ ± 7.67E^+05^	1.41E^+06^ ± 9.47E^+05^	1.61E^+06^ ± 8.03E^+05^	1.67E^+06^ ± 8.79E^+05^
TnC -/-	1.06E^+06^ ± 5.95E^+05^	1.19E^+06^ ± 7.01E^+05^	5.87E^+05^ ± 5.42E^+05^	8.21E^+05^ ± 4.46E^+05^

**Figure 6:**
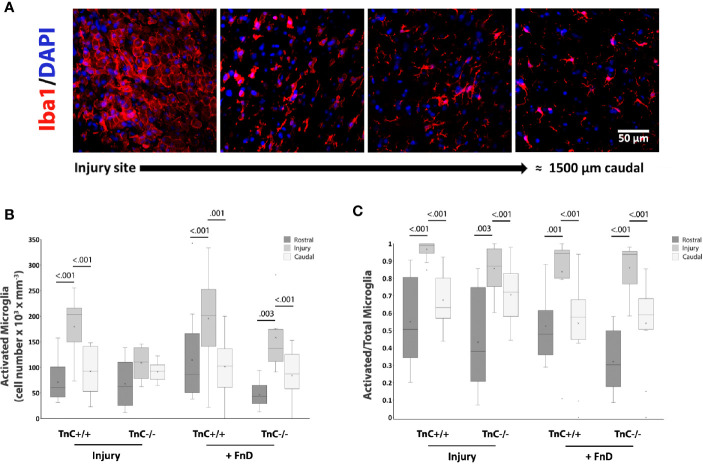
TnC affects activation of microglia in the injury region. **(A)** Representative images of Iba1+ and DAPI immunostaining show activated, polygonal microglia framed by astrocytes, at the injury site, and a more quiescent microglia phenotype further away. Calibration bar for all panels: 50 μm. **(B)** Density of activated microglia by stereo-investigator analysis at the injury region and surrounding rostral and caudal regions of equal size 7 days after injury. Results are shown as a box-and-whisker plot. The effects of genotype and sampling position on the density of activated microglia are significant (*p* < 0.001), as well as their interaction in the Injury group (*p* = 0.032) as analyzed by three-way ANOVA. The density of activated microglia in TnC+/+ mice in the injury region is higher in TnC+/+ mice in both Injury and FnD groups compared to the other sampling positions (*p* < 0.001). FnD application in TnC-/- mice increases the density of activated microglia at the injury region more so than rostrally (*p* = 0.003) or caudally (*p* < 0.001. **(C)** A box-and-whisker plot showing Activated/(Activated + Resting) microglia numbers per region. The proportion of activated microglia is higher at the injury site than rostrally and caudally in the injury and FnD groups of both genotypes (*p* < 0.001). n = 3 animals per group for each genotype.

Another typical glial scar element, especially within the first week after injury, are microglia/macrophages (40, 42). Whereas astrocyte processes overlap at the site of injury and cell bodies are difficult to distinguish individually, activated microglia/macrophages are recognized as distinct polygonal cells, being framed by astrocytes (**Figures 6A**). Thus, the density of activated and resting microglia/macrophages could be quantified throughout the injury region and surrounding rostral and caudal regions. The overall effects of genotype and sampling position on the density of activated microglia were significant (*p* < 0.001), as well as their interaction in the injury group (*p* = 0.032) (**Figure 6B**). The density of activated microglia/macrophages within the injury site was higher in TnC+/+ mice in both injury and FnD groups compared to other regions (*p* < 0.001). In FnD- treated TnC-/- mice, the density of activated microglia/macrophages in the injury region was higher than in rostral (*p* = 0.003) or caudal (*p* < 0.001) regions. When normalized to the total microglia/macrophage number, the proportion of activated microglia was higher in the injury site compared to rostral and to caudal positions in all of the conditions (*p* < 0.001) reaching the highest value in the TnC+/+ injury group (**Figure 6C**). Total microglial cell numbers were calculated as the sum of activated and resting microglia. Resting microglia density was a complementary image of activated microglia density (**Supplementary Figure S2**). Average numbers ± standard deviations of activated microglia in the spinal cord injury site of TnC +/+ and TnC -/- mice are presented in **Table 7**, and mean values ± standard deviations of the portion of activated microglia out of total microglia numbers is presented in **Table 8**.

**Table 7:** Average numbers ± standard deviations of activated out of total microglia in the injured spinal cord of TnC +/+ and TnC −/− mice (cell number x 10^3^ x mm^−3^).

	Injury			+FnD		
	Rostral	Injury	Caudal	Rostral	Injury	Caudal
TnC +/+	92.35 ± 42.79	179.70 ± 55.40	71.16 ± 39.33	101.69 ± 61.12	195.49 ± 80.70	114.49 ± 90.27
TnC-/-	91.44 ± 16.33	108.94 ± 30.62	67.95 ± 45.39	84.31 ± 47.43	158.27 ± 63.73	46.56 ± 23.80

**Table 8:** Mean values ± standard deviations of the portion of activated microglia numbers in the injured spinal cord of TnC +/+ and TnC −/− mice.

	Injury			+FnD		
	Rostral	Injury	Caudal	Rostral	Injury	Caudal
TnC +/+	0.68 ± 0.14	0.97 ± 0.05	0.55 ± 0.23	0.54 ± 0.27	0.84 ± 0.24	0.53 ± 19
TnC-/-	0.71 ± 0.15	0.86 ± 0.13	0.43 ± 0.28	0.54 ± 0.24	0.86 ± 0.14	0.32 ± 0.17

## Discussion

We studied the effects of TnC and its fragments on the glial reaction to injury *in vitro* and *in vivo*. In the context of an astrocyte scratch assay, we found that, independent of the genotype, TnC fragments attenuated gap closure. FnD and its combination with the fragment FnA were the most effective. Proliferation of astrocytes *in vitro* was decreased upon application of alternatively spliced FnD, FnA, FnC, their combinations and constitutively expressed Fn6-8 and EGFL fragments. These results suggest that fragments delay gap closure through decreased astrocyte proliferation.

Previous observations indicate that astrocyte proliferation is enhanced in a scratch wound assay (30), while TnC is known to reduce proliferation of adult human astrocytes *in vitro* (16). Alternatively spliced region encompassing FnIII repeats from A to D binds to the cell surface annexin II receptor (43), which inhibits cell migration in the process of gap closure (44). A smaller, 190 kDa isoform of TnC, comprising only the constitutively expressed domains, binds to contactin/F11 through FnIII repeats (45) and promotes cell adhesion (46). FnIII domain can also bind to the β subunit of integrin receptors (47, 48) and affect both cell migration and proliferation through reorganization of the actin cytoskeleton. (39, 49)

A scratch wound assay elicits *in vitro* a response of a single cell type, such as astrocytes, to mechanical injury only, with astrocytes tending to close the gap through migration and proliferation. On the other hand, *in vivo*, injury includes mechanical and chemical stages, as well as heterogenous cell types, disruption of the blood brain barrier and inflammation, all contributing to a more complex reaction during glial scar formation. Seven days after SCI, pro-inflammatory cells form the lesion core, while astrocytes reside on the border of the lesion site, initially providing protection of the surroundings, and eventually forming the scar tissue (40).

Levels of pro-inflammatory cytokines *Tnf-α* and *Il-1β* were increased *in vitro* in astrocytes upon addition of EGFL, FnA, FnD, and Fn6-8 fragments, regardless of the genotype. TnC has three main binding partners in inflammatory signalling: Toll-like receptor 4 (TLR4), integrins α9β1 and αVβ3. When TLR4 is activated by TnC, the most frequent outcome is the production of soluble pro-inflammatory mediators, such as IL-6, TNF-α and IL-1β by various cell types (39). These cytokines are upregulated also in peritoneal macrophages *via* activation by TnC of integrin αVβ3 and NFκB signaling pathways (49).

In our experiments, TnC-/- astrocyte cultures exhibited consistently higher GFAP protein and mRNA expression levels, regardless of the type of fragment used for treatment. As already reported, the GFAP expression level was higher in spinal cords of TnC-/- embryos, which is not seen thereafter in adult spinal cords of non-injured mice (38). In our study, *in vivo*, 7 days after SCI, GFAP immunoreactivity was the same in TnC+/+ and TnC-/- mice treated with vehicle or FnA after injury. Injection of FnD or Fn(D+A) led to a difference between the two genotypes, with higher levels in TnC+/+ than in TnC-/- mice (40).

*In vivo* proliferation at the injury site of the spinal cord was the same in TnC+/+ and TnC-/- mice 7 days after SCI. Since *in vitro* proliferation of astrocytes decreased upon fragment application, regardless of the genotype, it is thus possible that FnD and FnA induce proliferation of cell types other than astrocytes or that TnC does not affect cell proliferation in the tissue during this time frame after injury. We propose that this difference is due to different binding partners on the surface of different cell types.

TnC had been implicated in regulating production of pro-inflammatory cytokines/chemokines, chemotaxis and phagocytosis through the interaction with TLR4 in cultures of microglia (50). Our results on the microglia/macrophage response to injury showed that the FnD fragment increases the number of activated microglia/macrophages at the injury site.

Matrix metalloproteases (MMP) induce TnC turnover (46) cleaving it mostly within the alternatively spliced domains, a process through which they also generate soluble fragments which might have different functions than the whole TnC protein. In the present study, the FnD fragment was singled out as the most potent domain in mediating TnC effects on glial cells within the first week after SCI. Our results may explain how the astrocyte reaction is delayed and restricted to the border of the injury site to allow microglia/macrophages to form a lesion core during the first stages of glial scar formation through action of TnC and, in particular, FnD. Since this stage is generally regarded as a potential window for therapeutical approaches, modulation of TnC isoforms and their interaction partners could be considered to be valuable targets. Further research is needed to dissect the heterophilic interaction partners which mediate the effects of TnC fragments on astrocytes and microglia/macrophages upon injury.

## Data Availability Statement

The original contributions presented in the study are included in the article/**Supplementary Material**. Further inquiries can be directed to the corresponding authors.

## Ethics Statement

The animal study was reviewed and approved by the Ethics Committee of the Faculty of Biology, University of Belgrade.

## Author Contributions

DB performed the *in vitro* experiments, assisted in the *in vivo* experiment data analysis, and wrote the manuscript. MA assisted in the *in vitro* experiments, data analyses, and manuscript writing. MP assisted in the *in vivo* experiments, immunohistochemistry, and data analysis. IJ conceptualized and design the study, performed the *in vivo* experiments, and wrote the manuscript. EF provided study consultation and wrote the manuscript. MS provided encouragement for the study concept, envisioned the importance of tenascin-C domains, provided study consultation, and wrote the manuscript. PA provided the study concept and design, consultation, and wrote the manuscript. All authors contributed to the article and approved the submitted version.

## Funding

The study was funded by the DAAD/MESTD project “Involvement of Tenascin-C in Astrocyte Scarring After Spinal Cord Injury” (451-03-01766/2014-09/6) and the Ministry of Education, Science and Technological Development of the Republic of Serbia, contract number: 451-03-68/2020-14/200178.

## Conflict of Interest

The authors declare that the research was conducted in the absence of any commercial or financial relationships that could be construed as a potential conflict of interest.

## Supplementary Material

Supplementary Figure 1 Gap closure in astrocyte cultures of TnC+/+ and TnC-/- mice in the presence of different TnC fragments. Combined clustered stacked column chart displaying the relative wound closure at different time points after scratching and application of TnC fragments. Stacked columns represent the mean values of relative wound closure ± SD. n = 3 independent astrocyte culture preparations.

Supplementary Figure 2 Density of resting microglia within the spinal cord injury region in TnC+/+ and TnC-/- mice. Box plots show the density of resting microglia within the injury region, as well as in the surrounding rostral and caudal areas obtained 7 days after the spinal cord injury. Iba1 immunolabeling was assessed by stereological analysis. n = 3 animals per group for each genotype.
