# Corrigendum: Different Functions of Recombinantly Expressed Domains of Tenascin-C in Glial Scar Formation

**DOI:** 10.3389/fimmu.2021.672476

**Published:** 2021-03-16

**Authors:** Dunja Bijelić, Marija Adžić, Mina Perić, Igor Jakovčevski, Eckart Förster, Melitta Schachner, Pavle R. Andjus

**Affiliations:** ^1^ Centre for Laser Microscopy, Faculty of Biology, Institute of Physiology and Biochemistry “Jean Giaja”, University of Belgrade, Belgrade, Serbia; ^2^ Institut für Neuroanatomie und Molekulare Hirnforschung, Ruhr-Universität Bochum, Bochum, Germany; ^3^ Keck Center for Collaborative Neuroscience and Department of Cell Biology and Neuroscience, Rutgers University, Piscataway, NJ, United States

**Keywords:** astrocyte, glial scar, microglia/macrophages, spinal cord injury, tenascin-C

In the original article, there was a mistake in **Figure 2** as published. Instead of micrograph “+ FnA”, the micrograph for “+ Fn(D+A)” treatment in TnC +/+ genotype was duplicated by mistake. We have inserted the correct micrograph for “+ FnA”. The corrected **Figure 2** appears below.

**Figure 2:**
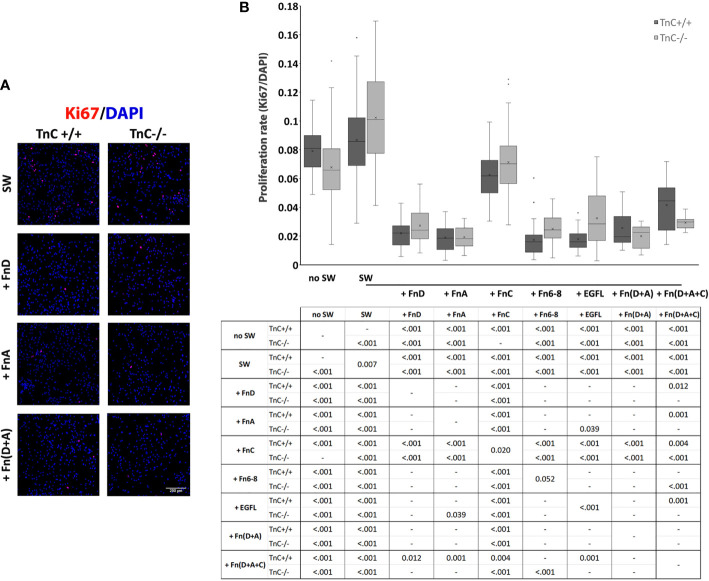
TnC fragments reduce proliferation in the astrocyte scratch wound assay. **(A)** Representative micrographs of Ki67+/DAPI+ immunofluorescence at 24 h after scratching in the control group and groups treated with FnA, FnD, and Fn(D+A); bar: 200 µm. **(B)** Proliferation was calculated as the number of Ki67+ nuclei compared to total DAPI+ nuclei. Results are presented as a box-and-whisker plot. Two-way ANOVA analysis shows a statistically significant interaction between the effects of genotype and treatment on cell proliferation rate (*p* = 0.005) with both the effects of genotype and treatment being significant (*p* = 0.040, *p* < 0.001, respectively). A statistically significant decrease in proliferation is seen in the presence of FnA, FnD, and Fn(D+A). All statistically significant pairwise comparisons are displayed below the box-and-whisker plot. n=3 independent astrocyte cultures.

The authors apologize for this error and state that this does not change the scientific conclusions of the article in any way. The original article has been updated.

