# Supplementary figures and images for: Different Functions of Recombinantly Expressed Domains of Tenascin-C in Glial Scar Formation
Source: Front Immunol. 2021 Feb 19;11:624612. doi: 10.3389/fimmu.2020.624612 (PMC7934619; doi:10.3389/fimmu.2020.624612)

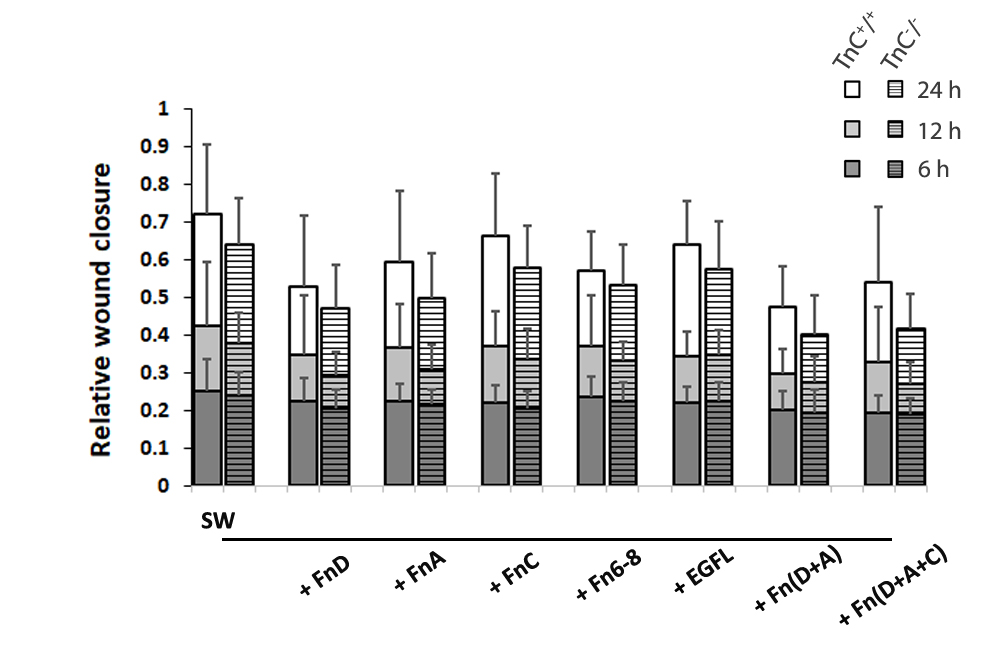

Supplement: Supplementary Figure 1 — Gap closure in astrocyte cultures of TnC+/+ and TnC-/- mice in the presence of different TnC fragments. Combined clustered stacked column chart displaying the relative wound closure at different time points after scratching and application of TnC fragments. Stacked columns represent the mean values of relative wound closure ± SD. n = 3 independent astrocyte culture preparations. [file Image_1.jpeg]

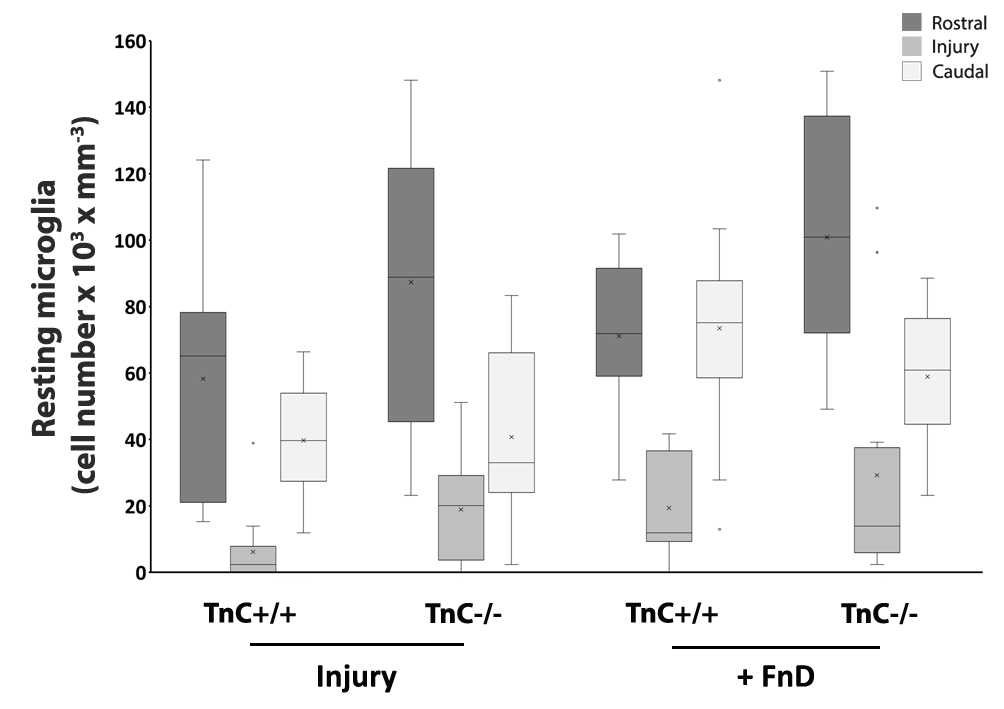

Supplement: Supplementary Figure 2 — Density of resting microglia within the spinal cord injury region in TnC+/+ and TnC-/- mice. Box plots show the density of resting microglia within the injury region, as well as in the surrounding rostral and caudal areas obtained 7 days after the spinal cord injury. Iba1 immunolabeling was assessed by stereological analysis. n = 3 animals per group for each genotype. [file Image_2.jpeg]
